# Age exerts differential effects on MRI misclassification for medial and lateral meniscal tears

**DOI:** 10.1002/jeo2.70797

**Published:** 2026-06-15

**Authors:** Paula Viza Gomes, Timothy Wee Shang Kouo, Santita Ebangwese, Julia Elizabeth Ralph, Madelynne Olexa, Gustavo Sosa Macias, John Twomey‐Kozak, Brian Chei‐Fai Lau

**Affiliations:** ^1^ Department of Orthopedic Surgery Duke University Hospital Durham North Carolina USA; ^2^ The Anne Burnett Marion School of Medicine at Texas Christian University Fort Worth Texas USA

**Keywords:** age factors, diagnostic accuracy, false negative, false positive, magnetic resonance imaging (MRI), meniscus

## Abstract

**Purpose:**

The influence of age on magnetic resonance imaging (MRI) misclassification patterns remains incompletely defined. The purpose of this study was to evaluate the association between patient age and MRI diagnostic performance by meniscus compartment, with a focus on error subtype.

**Methods:**

A retrospective diagnostic accuracy study was performed on 218 patients with intact anterior cruciate ligaments (ACLs) who underwent arthroscopic meniscal surgery between 2020 and 2024 with available preoperative MRI. The primary outcomes were compartment‐specific MRI diagnostic errors, including false‐positive and false‐negative findings, as well as sensitivity and specificity. Univariable logistic regression modelled the association between age (per 10‐year increase) and MRI misclassification outcomes, reported as odds ratios (ORs) with 95% confidence intervals (CIs).

**Results:**

A total of 218 knees were analysed for both medial and lateral menisci (mean age 46.4 ± 16.3 years; 50.5% male). MRI for detection of lateral meniscal tears demonstrated sensitivity of 0.44 (95% CI, 0.34–0.54) and specificity of 0.57 (95% CI, 0.49–0.66). MRI for detection of medial meniscal tears demonstrated higher sensitivity (0.75; 95% CI, 0.68–0.81) but lower specificity (0.27; 95% CI, 0.17–0.40). With increasing age, sensitivity of MRI for lateral tears declined while specificity increased; sensitivity for medial tears increased while specificity remained low. Each 10‐year increase in age was associated with higher odds of lateral false negatives (OR 1.32; 95% CI, 0.99–1.80), although this did not reach statistical significance, and significantly lower odds of medial false negatives (OR 0.48; 95% CI, 0.36–0.62). Receiver operating characteristic analysis showed moderate discrimination for age (area under the curve 0.62–0.80), with thresholds near 46 years.

**Conclusion:**

Increasing age was associated with fewer missed medial meniscal tears but more missed lateral tears, with fewer false‐positive lateral MRI findings. These patterns may reflect degenerative medial signal changes and lower prevalence of traumatic lateral tears in older patients. Incorporating age with clinical findings may improve compartment‐specific MRI interpretation.

**Level of Evidence:**

Level III.

**Abbreviations**
AUC: area under the curve
CI: confidence interval
FN: false negative
FP: false positive
MRI: magnetic resonance imaging
NPV: negative predictive value
OR: odds ratio
PPV: positive predictive value
ROC: receiver operating characteristic
TN: true negative
TP: true positive

## INTRODUCTION

Meniscal tears are among the most common causes of knee pain and dysfunction across the adult lifespan [7]. Physical exam manoeuvres alone are often insufficient in diagnosing meniscal pathology and tear patterns [17, 19, 25], and therefore magnetic resonance imaging (MRI) is widely used as the primary noninvasive modality for evaluating suspected meniscal pathology and guiding clinical decision‐making [22]. Numerous studies have demonstrated high diagnostic accuracy of MRI for detecting meniscal tears, with reported sensitivity ranging from approximately 78%–92% and specificity from 88% to 95%, depending on whether the medial or lateral meniscus is involved [4, 8, 15, 22].

Despite this overall accuracy, MRI performance may vary across clinical contexts and patient populations [5, 10, 12, 14, 24]. Patient age may represent another important factor influencing MRI interpretation, with prior work suggesting MRI diagnostic accuracy may decline with advancing age, with negative correlations reported between patient age and diagnostic accuracy for medial meniscal tears and articular cartilage damage [7, 8, 11]. The increasing prevalence of degenerative meniscal signal abnormalities in older adults may therefore complicate MRI interpretation and reduce diagnostic specificity [7, 11].

However, the medical literature contains limited age‐stratified diagnostic accuracy data for MRI evaluation of meniscal tears. Most studies have defined 'older' populations using single age cutoffs, commonly 50 years or older, rather than examining age as a continuous or ordinal variable across multiple age decades [8, 18]. As a result, it remains unclear whether age‐related differences in MRI accuracy reflect true diagnostic limitations or the increasing prevalence of degenerative and asymptomatic meniscal pathology that complicates image interpretation.

Therefore, the purpose of this study was to evaluate the association between patient age and MRI diagnostic performance for medial and lateral meniscal tears using arthroscopic findings as the reference standard, with particular attention to overall misclassification and error subtype. We hypothesised that increasing age would be associated with distinct, compartment‐specific patterns of MRI misclassification, reflecting differences in degenerative pathology and tear morphology between the medial and lateral menisci.

## METHODS

### Study design and population

This retrospective diagnostic accuracy study evaluated the association between patient age and the accuracy of preoperative MRI, using intraoperative arthroscopy as the reference standard. Patients who underwent knee arthroscopy at a single tertiary care centre from 2020 to 2024 were retrospectively identified. All procedures were performed by a fellowship‐trained sports medicine orthopedic surgeon (BCL). Patients with available preoperative MRI and documented intraoperative meniscal assessment were included. The medial and lateral menisci were analysed separately. For each compartment, MRI findings were compared with arthroscopic findings and classified as true positive (TP), true negative (TN), false positive (FP) or false negative (FN).

### Primary outcome

The primary outcome of interest was the binary presence or absence of meniscal tear within each compartment (medial and lateral), as determined by arthroscopy. Tear morphology (e.g., radial, horizontal, complex and root), and degree of meniscal degeneration were not separately analysed. This approach was chosen to allow consistent classification across operative reports and to focus specifically on compartment‐level diagnostic performance and misclassification patterns, independent of tear subtype heterogeneity.

### Statistical analysis

Diagnostic performance was assessed separately for the medial and lateral menisci using sensitivity, specificity, positive predictive value (PPV), negative predictive value (NPV), and overall accuracy, each with 95% confidence intervals (CIs) (Wilson method). For descriptive analyses, diagnostic metrics were stratified by age group (<30, 30–44, 45–59, and ≥60 years). Age was modelled continuously (per 10‐year increase) in all inferential analyses. Logistic regression was used to evaluate associations between age and MRI misclassification. Separate models were constructed for false‐negative outcomes among arthroscopically confirmed tears and false‐positive outcomes among arthroscopically confirmed non‐tears, stratified by meniscal compartment. Odds ratios (ORs) with 95% CIs were reported. Potential nonlinear associations between age and misclassification were explored using natural cubic spline logistic regression and segmented (piecewise) logistic regression. Receiver operating characteristic (ROC) analysis was additionally performed as an exploratory approach to assess the ability of age to stratify risk of MRI misclassification. Note that this application of ROC analysis evaluates age as a continuous predictor variable rather than assessing the diagnostic accuracy of MRI itself. Area under the curve (AUC) values, Youden‐derived exploratory thresholds, sensitivity, and specificity were reported. All analyses were performed using R version 4.4.3 (R Foundation for Statistical Computing, Vienna, Austria). Statistical significance was defined as a two‐sided *p*‐value < 0.05.

All analyses were performed using R version 4.4.3 (R Foundation for Statistical Computing, Vienna, Austria). Statistical significance was defined as a two‐sided *p*‐value < 0.05.

## RESULTS

### Patient demographics and characteristics

A total of 218 patients were included in the analysis (Table 1). Of these, 110 (50.5%) were male, and 114 (52.3%) underwent surgery on the left knee and the mechanism of injury was atraumatic in 55.00% of cases. Group sizes: <30 (*n* = 42), 30–44 (*n* = 47), 45–59 (*n* = 79), ≥60 (*n* = 50). Observed ranges for open‐ended strata were 13–29 and 61–79 years, respectively.

**Table 1:** Patient demographics and characteristics.

Characteristic	Value
Number of patients	218
Age at surgery, mean ± SD (years)	46.4 ± 16.3
Body mass index, mean ± SD (kg/m^2^)	30.3 ± 6.5
Male sex, *n* (%)	110 (50.5%)
Left knee surgery, *n* (%)	114 (52.3%)
Mechanism of injury, *n* (%)	
Atraumatic	120 (55.0%)
Athletic	51 (23.4%)
Occupational	20 (9.2%)
Traumatic	27 (12.5%)

Abbreviation: SD, standard deviation.

### Overall diagnostic performance

For the lateral meniscus, MRI demonstrated modest diagnostic performance. There were 39 true positives, 74 true negatives, 55 false positives and 50 false negatives. Sensitivity was 0.438 (95% CI, 0.340–0.542), and specificity was 0.574 (95% CI, 0.487–0.656). The positive predictive value was 0.415, and the negative predictive value was 0.597 (Table 2). For the medial meniscus, MRI demonstrated substantially higher sensitivity but markedly lower specificity. There were 121 true positives, 15 true negatives, 41 false positives, and 41 false negatives. Sensitivity was 0.747 (95% CI, 0.675–0.808), whereas specificity was 0.268 (95% CI, 0.170–0.396). The positive predictive value was 0.747 (Table 2). The low specificity reflects frequent MRI overcalling in arthroscopically confirmed medial non‐tear cases.

**Table 2:** Overall diagnostic performance of MRI for lateral and medial meniscal tears.

Meniscus	TP	TN	FP	FN	Sensitivity (95% CI)	Specificity (95% CI)	PPV (95% CI)	NPV (95% CI)	Accuracy (95% CI)
Lateral	39	74	55	50	0.44 (0.34–0.54)	0.57 (0.49–0.66)	0.41 (0.32–0.52)	0.60 (0.51–0.68)	0.52 (0.45–0.58)
Medial	121	15	41	41	0.75 (0.67–0.81)	0.27 (0.17–0.40)	0.75 (0.67–0.81)	0.27 (0.17–0.40)	0.62 (0.56–0.69)

Abbreviations: CI, confidence interval; FN, false negative; FP, false positive; MRI, magnetic resonance imaging; NPV, negative predictive value; PPV, positive predictive value; TN, true negative; TP, true positive.

Age‐stratified analyses demonstrated distinct age‐related patterns in MRI performance (Table 3). For the lateral meniscus, MRI sensitivity was similar in younger patients (<30 years, 0.545; 30–44 years, 0.565) and lower in the older age strata (45–59 years, 0.353; ≥60 years, 0.381). In contrast, lateral specificity increased with age, rising from 0.259 in patients younger than 30 years to 0.614 in patients aged 30–44 years and 0.610 in patients aged 45–59 years, then further increasing to 0.758 in patients aged 60 years or older. For the medial meniscus, MRI sensitivity increased substantially with age, rising from 0.375 in patients younger than 30 years to 0.703 in patients aged 30–44 years, 0.865 in patients aged 45–59 years, and 0.927 in patients aged 60 years or older (Table 3).

**Table 3:** Age‐stratified diagnostic performance of MRI for medial and lateral meniscal tears.

Meniscus	Age group (years)	TP	TN	FP	FN	Sensitivity (95% CI)	Specificity (95% CI)	PPV (95% CI)	NPV (95% CI)	Accuracy (95% CI)
Lateral	<30	6	7	20	5	0.55 (0.28–0.79)	0.26 (0.13–0.45)	0.23 (0.11–0.42)	0.58 (0.32–0.81)	0.34 (0.21–0.50)
	30–44	13	17	11	10	0.57 (0.37–0.74)	0.61 (0.42–0.76)	0.54 (0.35–0.72)	0.63 (0.44–0.78)	0.59 (0.45–0.71)
	45–59	12	25	16	22	0.35 (0.21–0.52)	0.61 (0.46–0.74)	0.43 (0.27–0.61)	0.53 (0.39–0.67)	0.49 (0.38–0.60)
	≥60	8	25	8	13	0.38 (0.21–0.59)	0.76 (0.59–0.87)	0.50 (0.28–0.72)	0.66 (0.50–0.79)	0.61 (0.48–0.73)
Medial	<30	12	2	4	20	0.38 (0.23–0.55)	0.33 (0.10–0.70)	0.75 (0.51–0.90)	0.09 (0.03–0.28)	0.37 (0.23–0.53)
	30–44	26	6	8	11	0.70 (0.54–0.83)	0.43 (0.21–0.67)	0.76 (0.60–0.88)	0.35 (0.17–0.59)	0.63 (0.49–0.75)
	45–59	45	3	20	7	0.87 (0.75–0.93)	0.13 (0.05–0.32)	0.69 (0.57–0.79)	0.30 (0.11–0.60)	0.64 (0.53–0.74)
	≥60	38	4	9	3	0.93 (0.81–0.97)	0.31 (0.13–0.58)	0.81 (0.67–0.90)	0.57 (0.25–0.84)	0.78 (0.65–0.87)

Abbreviations: CI, confidence interval; FN, false negative; FP, false positive; MRI, magnetic resonance imaging; NPV, negative predictive value; PPV, positive predictive value; TN, true negative; TP, true positive.

### Age as a predictor of MRI misclassification

To evaluate the effect of age on diagnostic accuracy independent of disease prevalence, logistic regression models and analysis were constructed separately for false‐negative and false‐positive outcomes. Among patients with arthroscopically confirmed lateral meniscus tears, increasing age was associated with higher odds of a false‐negative MRI. Each 10‐year increase in age was associated with a 32% increase in the odds of a false‐negative result; however, this association did not reach statistical significance (OR, 1.32; 95% CI, 0.99–1.80; *p* = 0.063). In contrast, among patients with no lateral meniscus tear identified at arthroscopy, including those with isolated medial meniscus tears or no meniscal tear, increasing age was associated with significantly lower odds of a false‐positive MRI. Each 10‐year increase in age reduced the odds of a false‐positive interpretation by 33% (OR, 0.67; 95% CI, 0.53–0.83; *p* < 0.001). In patients with arthroscopically confirmed medial meniscus tears, increasing age was strongly associated with a reduced risk of false‐negative MRI. Each 10‐year increase in age was associated with a 52% reduction in the odds of a false‐negative result (OR, 0.48; 95% CI, 0.36–0.62; *p* < 0.001) (Table 4). In contrast, age was not significantly associated with false‐positive MRI interpretation (OR, 1.39 per 10 years; 95% CI, 0.93–2.14; *p* = 0.12).

**Table 4:** Association between age and MRI misclassification by meniscal compartment.

Meniscus	Outcome	Odds ratio (per 10 years)	95% CI	*p*‐value
Lateral	False negative (true tear)	1.32	0.99–1.80	0.063
Lateral	False positive (true no‐tear)	0.67	0.53–0.83	<0.001
Medial	False negative (true tear)	0.48	0.36–0.62	<0.001
Medial	False positive (true no‐tear)	1.39	0.93–2.14	0.12

Abbreviations: CI, confidence interval; MRI, magnetic resonance imaging.

### Nonlinear age effects and fall‐off patterns

**Figure 1:**
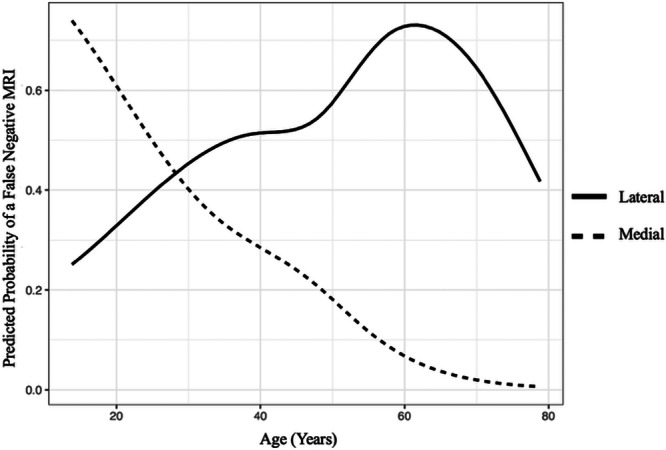
Predicted probability of false‐negative MRI diagnosis across age for medial and lateral menisci. MRI, magnetic resonance imaging.

**Figure 2:**
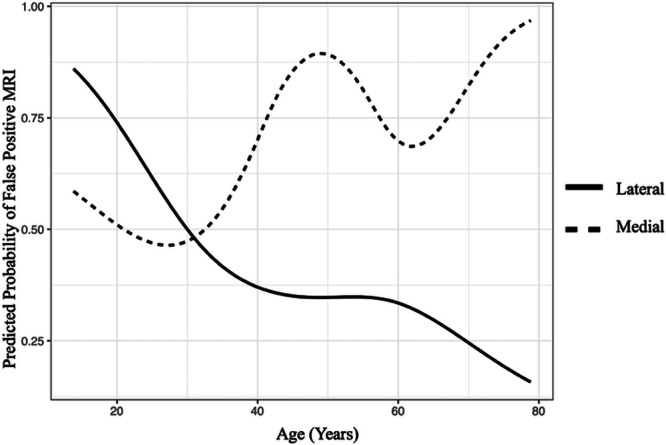
Predicted probability of false‐positive MRI diagnosis across age for medial and lateral menisci. MRI, magnetic resonance imaging.

Spline‐based logistic regression models demonstrated nonlinear relationships between age and MRI misclassification risk (Figures 1 and 2). For the medial meniscus, the predicted probability of a false‐negative MRI declined progressively with increasing age, whereas for the lateral meniscus, the predicted probability of a false‐negative MRI increased with age (Figure 1). False‐positive probabilities demonstrated an inverse pattern, particularly for the lateral compartment, in which younger age was associated with a higher likelihood of MRI overcalling (Figure 2). These spline analyses were used to visualise age‐related trends and were not interpreted as inferential models.

### ROC analysis and age thresholds for misclassification risk

**Figure 3:**
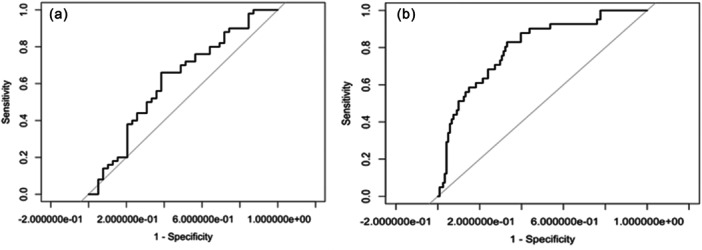
Receiver operating characteristics (ROC) curves for patient age predicting false‐negative MRI by meniscus compartment. (a) Lateral meniscal tear, (b) medial meniscal tears. Sensitivity is plotted against 1 − specificity. The diagonal line represents the line of no discrimination. MRI, magnetic resonance imaging.

Receiver operating characteristic analysis was performed to explore age thresholds associated with MRI misclassification risk (Table 5 and Figure 3). For false‐negative MRI among lateral meniscus tears, age demonstrated modest discriminative ability, with an AUC of 0.619 (95% CI, 0.499–0.739). The optimal Youden cutoff occurred at 46.5 years, corresponding to a sensitivity of 0.66 and a specificity of 0.62 (Table 5). For false‐negative MRI among medial meniscus tears, age demonstrated stronger discriminative ability, with an AUC of 0.799 (95% CI, 0.722–0.876), and an optimal Youden cutoff of 46.3 years, corresponding to a sensitivity of 0.83 and a specificity of 0.67 (Table 5).

**Table 5:** ROC analysis of age as a predictor of MRI misclassification.

Meniscus	Outcome	AUC (95% CI)	Youden age cutoff (years)	Sensitivity	Specificity
Lateral	False negative (true tear)	0.619 (0.499–0.739)	46.5	0.66	0.62
	False positive (true no‐tear)	0.679 (0.584–0.774)	35.1	0.47	0.85
Medial	False negative (true tear)	0.799 (0.722–0.876)	46.3	0.83	0.67
	False positive (true no‐tear)	0.624 (0.441–0.808)	44.6	0.71	0.60

Abbreviations: AUC, area under the curve; CI, confidence interval; MRI, magnetic resonance imaging; ROC, receiver operating characteristic.

For false‐positive MRI, age predicted misclassification with an AUC of 0.679 (95% CI, 0.584–0.774) for the lateral meniscus, with a Youden cutoff of 35.1 years. For the medial meniscus, age predicted false‐positive MRI with an AUC of 0.624 (95% CI, 0.441–0.808), with a Youden cutoff of 44.6 years (Table 5). Segmented logistic regression identified age breakpoints at which the slope of MRI misclassification risk changed for both false‐negative and false‐positive outcomes in each meniscal compartment (Supporting Information: Table S1 and Figure S1).

## DISCUSSION

This present study found that patient age exerts a differential effect on MRI diagnostic reliability, defined here as the rate of false‐positive and false‐negative MRI interpretations relative to arthroscopic findings, when comparing medial versus lateral meniscal tears. Increasing age was associated with reduced MRI misclassification for medial meniscal tears, driven by a substantial decrease in false‐negative findings that outweighed a modest, non‐significant increase in false‐positive interpretations. This pattern was further supported by ROC analysis in which age demonstrated its strongest, albeit modest, discriminatory ability for this specific outcome (AUC 0.799). In contrast, for the lateral meniscus, increasing age was significantly associated with reduced false‐positive findings, while false‐negative findings demonstrated a non‐significant trend in the opposite direction, suggesting that age‐related changes shift the balance of diagnostic error in opposing directions across the two compartments.

Prior histologic–MRI correlation studies have established that Grade 3 intra‐meniscal signal intensity and diffuse degenerative signal patterns are common in elderly menisci, occurring in both torn and intact structures, thereby reducing MRI specificity for true tears [8, 9, 18]. These degeneration‐related alterations generate increased intra‐meniscal signal that can mimic tear morphology and lower the interpretive threshold for diagnosing a tear on MRI [1, 2, 13], as the rising prevalence of degenerative changes with advancing age alters the overall context in which meniscal imaging is interpreted [4, 8, 20]. These prior findings provide a plausible explanation for the increase in false‐positive MRI interpretations for medial meniscal pathology observed with advancing age in the present study.

This same degenerative environment may also contribute to the significant reduction in false‐negative MRI findings observed for medial meniscal pathology with increasing age, as the ubiquity of degenerative signal changes in older menisci may lower the interpretive threshold for classifying a tear, making it less likely that true pathology is dismissed as a normal variant. This phenomenon is consistent with prior work demonstrating that MRI sensitivity remains high in older adults when equivocal findings are classified as tears, albeit at the expense of specificity [3, 6]. Subhas et al. showed that while specificity for medial meniscal tears declines substantially when indeterminate findings are included, sensitivity approaches near‐perfect levels, reflecting a shift in diagnostic error toward false‐positive rather than false‐negative interpretations [18]. Prior longitudinal work has demonstrated that meniscal degeneration carries prognostic significance, as it is associated with structural progression of knee disease [21, 23]. Ward et al. reported that patients with meniscal degeneration had up to fivefold higher odds of developing accelerated knee osteoarthritis or destabilising meniscal tears, underscoring the prognostic significance of accurately identifying degenerative meniscal changes on MRI, particularly in older patients [23]. Thus, the observed shift toward fewer false‐negative and more false‐positive interpretations in older patients may reflect the biological and structural consequences of age‐related meniscal degeneration on radiologic interpretation, rather than a purely detrimental loss of diagnostic specificity.

In contrast, the lateral meniscus appears to follow a different age‐related pattern. Lateral meniscal tears are less strongly associated with chronic degenerative processes and are more commonly related to acute injury mechanisms, often occurring in younger athletes [16]. As the prevalence of traumatic lateral meniscal tears decreases with advancing age, radiologists may apply a higher diagnostic threshold when interpreting lateral meniscal abnormalities, which could explain the observed significant reduction in false‐positive findings with increasing age. However, this same interpretive threshold may contribute to subtle or atypical lateral meniscal tears being overlooked, resulting in a non‐significant trend toward increased false‐negative MRI findings with advancing age. Prior work reporting higher MRI specificity but lower sensitivity for lateral meniscal tears supports the presence of this diagnostic tradeoff [22]. Together, these opposing age‐related effects illustrate that gains in lateral meniscal specificity with advancing age may come at the expense of sensitivity, particularly in patients in the fifth decade of life and beyond (Table 5).

Overall, these findings are clinically relevant as they suggest that MRI interpretation for suspected meniscal pathology should be considered in the context of both patient age and meniscal compartment. In older patients, medial meniscal abnormalities on MRI may be less likely to represent missed pathology and more likely to reflect degenerative or broadly characterised tear patterns, whereas subtle lateral meniscal tears may remain difficult to detect. Conversely, in younger patients, lateral meniscal MRI abnormalities may be more prone to overinterpretation. Incorporating patient age into MRI interpretation may therefore help clinicians better contextualise diagnostic uncertainty and align imaging findings with clinical decision‐making.

In practice, these patterns may offer useful context when clinical and imaging findings are not fully concordant. For instance, a medial meniscal abnormality in a middle‐aged or older patient may be more likely to reflect degenerative change, while a normal‐appearing lateral meniscus in the same population may not fully exclude pathology when clinical suspicion persists. These observations are hypothesis‐generating, and the age‐related patterns described here should not be used to guide clinical decisions or establish diagnostic thresholds until prospective, multi‐centre validation has been performed.

This study has several important limitations. This was a retrospective single‐centre study performed at a tertiary referral institution, which may limit generalisability to other practice settings. All arthroscopic procedures were performed by a single fellowship‐trained sports medicine surgeon, which enhances internal consistency but may reduce external validity. Additionally, MRI interpretations were not standardised or re‐reviewed for the purposes of this study. Instead, original clinical radiology reports were used. Therefore, inter‐reader variability and differences in radiologist experience could not be accounted for. Although arthroscopy was used as the reference standard, only patients who ultimately underwent surgery were included. This introduces potential selection bias, as the cohort likely represents a surgically enriched population with higher pretest probability of meniscal pathology. Patients with negative MRIs who did not proceed to surgery were not captured, which may influence estimates of diagnostic performance.

Additionally, the number of arthroscopically confirmed medial non‐tear cases was relatively small, resulting in wide CIs for medial specificity estimates and limiting statistical power for false‐positive modelling in the medial compartment. This imbalance may have affected precision of compartment‐specific comparisons. Furthermore, logistic regression models were univariable and conditioned only on age. Potential confounders such as body mass index, mechanism of injury, chronicity of symptoms, osteoarthritis severity, and concomitant ligamentous injury were not included in multivariable analyses. These factors may independently influence MRI performance and misclassification patterns.

Subdividing by tear type would have substantially reduced sample size within each age stratum, limiting statistical power, and therefore this present study focused specifically on presence or absence of tear by compartment. This study is subject to verification bias because only patients who underwent arthroscopy were included. Patients with negative or equivocal MRI findings who did not proceed to surgery were not captured, and arthroscopy was therefore available only in a selected subgroup of patients. This may inflate sensitivity and distort specificity relative to estimates that would be obtained in an unselected population undergoing MRI for suspected meniscal pathology. Furthermore, logistic regression models were univariable and evaluated age as the primary predictor of interest. Potential confounders such as body mass index, osteoarthritis severity, chronicity of symptoms, and other degenerative factors were not fully available in standardised form for multivariable modelling. All patients in this cohort had intact anterior cruciate ligaments (ACLs); therefore, concomitant ACL injury was not a source of confounding. Accordingly, the observed associations may reflect age‐related degenerative burden or correlated clinical factors rather than age as an isolated independent determinant of MRI performance.

Finally, MRI technique parameters (magnet strength, sequence protocol, slice thickness) were not analysed, and variability in imaging acquisition may have influenced diagnostic performance. Additionally, this study evaluated presence or absence of meniscal tear rather than tear morphology, tear chronicity, or degree of degeneration, which may further modulate age‐related diagnostic accuracy patterns.

## CONCLUSION

Patient age influences the diagnostic accuracy of MRI for meniscal tears in a compartment‐specific manner, altering the balance between false‐positive and false‐negative findings differently across compartments. These findings suggest that incorporating patient age and meniscal compartment into MRI interpretation may improve diagnostic accuracy and inform clinical decision‐making, though prospective validation is needed before age‐based thresholds can be formally applied.

## AUTHOR CONTRIBUTIONS

All authors contributed to the study conception and design. Material preparation and data collection were performed by Santita Ebangwese, Julia Elizabeth Ralph, Madelynne Olexa, Gustavo Sisa Macias, and Jack Twomey‐Kozac. Statistical analyses were performed by Timothy Kouo and Paula Viza Gomes. Interpretation of data was performed by Paula Viza Gomes, Timothy Kouo, and Brian Chei‐Fai Lau. The first draft of the manuscript was written by Paula Viza Gomes and Timothy Kouo. All authors commented on previous versions of the manuscript. All authors read and approved the final manuscript.

## FUNDING

The authors have no funding to report.

## CONFLICT OF INTEREST STATEMENT

Brian Chei‐Fai Lau: Consultant for Miach Orthopedics, Arthrex, Cytek, and Newclip, with royalties from Newclip.

## ETHICS STATEMENT

Please include the name of the institutional review board (IRB) and the approval number. If not applicable, please state so. Duke Health Institutional Review Board, IRB #:111722.

## Supporting information

Supporting information file 1.

## Data Availability

Encourages data sharing.

## References

[1] Abram SGF , Beard DJ , Price AJ . National consensus on the definition, investigation, and classification of meniscal lesions of the knee. Knee. 2018;25(5):834–840. 29983330 10.1016/j.knee.2018.06.001

[2] Ahmad R . Intra‐substance meniscal changes and their clinical significance: a meta‐analysis. Sci Rep. 2021;11(1):3642. 33574469 10.1038/s41598-021-83181-5 PMC7878874

[3] Alaia EF , Samim M , Khodarahmi I , Zech JR , Spath AR , Da Silva Cardoso M , et al. Utility of MRI for patients 45 years old and older with hip or knee pain: a systematic review. Am J Roentgenol. 2024;222(6):e2430958. 38568033 10.2214/AJR.24.30958

[4] Beaufils P , Becker R , Kopf S , Englund M , Verdonk R , Ollivier M , et al. Surgical management of degenerative meniscus lesions: the 2016 ESSKA meniscus consensus. Knee Surg Sports Traumatol Arthrosc. 2017;25(2):335–346. 28210788 10.1007/s00167-016-4407-4 PMC5331096

[5] De Smet AA , Norris MA , Yandow DR , Quintana FA , Graf BK , Keene JS . MR diagnosis of meniscal tears of the knee: importance of high signal in the meniscus that extends to the surface. Am J Roentgenol. 1993;161(1):101–107. 8517286 10.2214/ajr.161.1.8517286

[6] Deshpande BR , Losina E , Smith SR , Martin SD , Wright RJ , Katz JN . Association of MRI findings and expert diagnosis of symptomatic meniscal tear among middle‐aged and older adults with knee pain. BMC Musculoskelet Disord. 2016;17:154. 27067990 10.1186/s12891-016-1010-2 PMC4827168

[7] Duong V , Oo WM , Ding C , Culvenor AG , Hunter DJ . Evaluation and treatment of knee pain: a review. JAMA. 2023;330(16):1568–1580. 37874571 10.1001/jama.2023.19675

[8] Englund M , Guermazi A , Gale D , Hunter DJ , Aliabadi P , Clancy M , et al. Incidental meniscal findings on knee MRI in middle‐aged and elderly persons. N Engl J Med. 2008;359(11):1108–1115. 18784100 10.1056/NEJMoa0800777 PMC2897006

[9] Hodler J , Haghighi P , Pathria MN , Trudell D , Resnick D . Meniscal changes in the elderly: correlation of MR imaging and histologic findings. Radiology. 1992;184(1):221–225. 1609084 10.1148/radiology.184.1.1609084

[10] Kim SH , Lee HJ , Jang YH , Chun KJ , Park YB . Diagnostic accuracy of magnetic resonance imaging in the detection of type and location of meniscus tears: comparison with arthroscopic findings. J Clin Med. 2021;10(4):606. 33562787 10.3390/jcm10040606 PMC7914628

[11] Koch JEJ , Ben‐Elyahu R , Khateeb B , Ringart M , Nyska M , Ohana N , et al. Accuracy measures of 1.5‐tesla MRI for the diagnosis of ACL, meniscus and articular knee cartilage damage and characteristics of false negative lesions: a level III prognostic study. BMC Musculoskelet Disord. 2021;22(1):124. 33514358 10.1186/s12891-021-04011-3 PMC7847141

[12] Kuikka PI , Sillanpää P , Mattila VM , Niva MH , Pihlajamäki HK . Magnetic resonance imaging in acute traumatic and chronic meniscal tears of the knee: a diagnostic accuracy study in young adults. Am J Sports Med. 2009;37(5):1003–1008. 19218558 10.1177/0363546508329543

[13] Li CA , Kim MK , Kim IH , Lee JH , Jang KY , Lee SY . Correlation of histological examination of meniscus with MR images: focused on high signal intensity of the meniscus not caused by definite meniscal tear and impact on MR diagnosis of tears. Korean J Radiol. 2013;14(6):935–945. 24265570 10.3348/kjr.2013.14.6.935 PMC3835642

[14] Oei EHG , Nikken JJ , Verstijnen ACM , Ginai AZ , Myriam Hunink MG . MR imaging of the menisci and cruciate ligaments: a systematic review. Radiology. 2003;226(3):837–848. 12601211 10.1148/radiol.2263011892

[15] Phelan N , Rowland P , Galvin R , O'Byrne JM . A systematic review and meta‐analysis of the diagnostic accuracy of MRI for suspected ACL and meniscal tears of the knee. Knee Surg Sports Traumatol Arthrosc. 2016;24(5):1525–1539. 26614425 10.1007/s00167-015-3861-8

[16] Ridley TJ , McCarthy MA , Bollier MJ , Wolf BR , Amendola A . Age differences in the prevalence of isolated medial and lateral meniscal tears in surgically treated patients. Iowa Orthop J. 2017;37:91–94. 28852341 PMC5508267

[17] Rinonapoli G , Carraro A , Delcogliano A . The clinical diagnosis of meniscal tear is not easy. Reliability of two clinical meniscal tests and magnetic resonance imaging. Int J Immunopathol Pharmacol. 2011;24(1 Suppl 2):39–44. 21669136 10.1177/03946320110241S208

[18] Subhas N , Sakamoto FA , Mariscalco MW , Polster JM , Obuchowski NA , Jones MH . Accuracy of MRI in the diagnosis of meniscal tears in older patients. Am J Roentgenol. 2012;198(6):W575–W580. 22623573 10.2214/AJR.11.7226

[19] Sladjan T , Zoran V , Zoran B . Correlation of clinical examination, ultrasound sonography, and magnetic resonance imaging findings with arthroscopic findings in relation to acute and chronic lateral meniscus injuries. J Orthop Sci. 2014;19(1):71–76. 24141393 10.1007/s00776-013-0480-4

[20] Tsujii A , Nakamura N , Horibe S . Age‐related changes in the knee meniscus. Knee. 2017;24(6):1262–1270. 28970119 10.1016/j.knee.2017.08.001

[21] Villagran M , Driban JB , Lu B , MacKay JW , McAlindon TE , Harkey MS . Radiomic features of the medial meniscus predicts incident destabilizing meniscal tears: data from the osteoarthritis initiative. J Orthop Res. 2024;42(9):2080–2087. 38747030 10.1002/jor.25851 PMC11336561

[22] Wang W , Li Z , Peng HM , Bian YY , Li Y , Qian WW , et al. Accuracy of MRI diagnosis of meniscal tears of the knee: a meta‐analysis and systematic review. J Knee Surg. 2021;34(2):121–129. 31390675 10.1055/s-0039-1694056

[23] Ward RJ , Driban JB , MacKay JW , McAlindon TE , Lu B , Eaton CB , et al. Meniscal degeneration is prognostic of destabilzing meniscal tear and accelerated knee osteoarthritis: data from the osteoarthritis initiative. J Orthop Res. 2023;41(11):2418–2423. 37094976 10.1002/jor.25575 PMC10592659

[24] Wong KPL , Han AX , Wong JLY , Lee DYH . Reliability of magnetic resonance imaging in evaluating meniscal and cartilage injuries in anterior cruciate ligament‐deficient knees. Knee Surg Sports Traumatol Arthrosc. 2017;25(2):411–417. 27342983 10.1007/s00167-016-4211-1

[25] Yan R , Wang H , Ji Z , Guo Y . Predicted probability of meniscus tears: comparing history and physical examination with MRI. Swiss Med Wkly. 2011;141:w13314. 22180191 10.4414/smw.2011.13314

