# Supplementary material for: Age exerts differential effects on MRI misclassification for medial and lateral meniscal tears
Source: J Exp Orthop. 2026 Jun 15;13(2):e70797. doi: 10.1002/jeo2.70797 (PMC13266572; doi:10.1002/jeo2.70797)
Supplement: Supplementary file 1 — Supporting information file 1. [file JEO2-13-e70797-s001.docx]

**Appendix**

Table S1 . Segmented logistic regression–estimated age breakpoints for MRI misclassification

| **Meniscus** | **Outcome** | **Estimated breakpoint age (years)** |
| --- | --- | --- |
| Lateral | False negative (true tear) | 19.5 |
|  | False positive (true no-tear) | 37.4 |
| Medial | False negative (true tear) | 17.9 |
|  | False positive (true no-tear) | 48.2 |

Figure S1: Segmented logistic regression plots illustrating the relationship between patient age and MRI misclassification of meniscal tears.


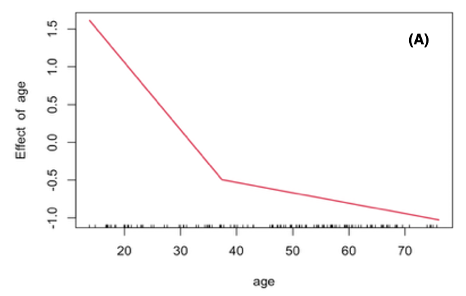


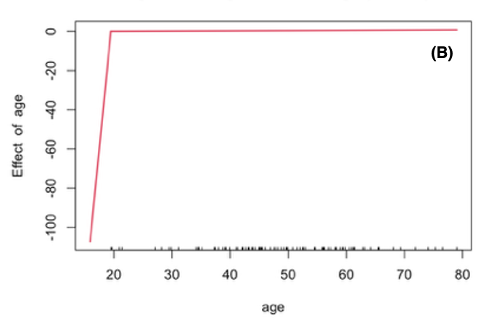


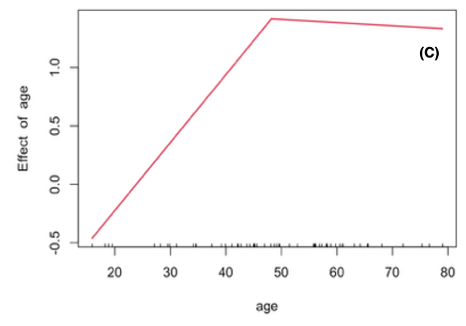


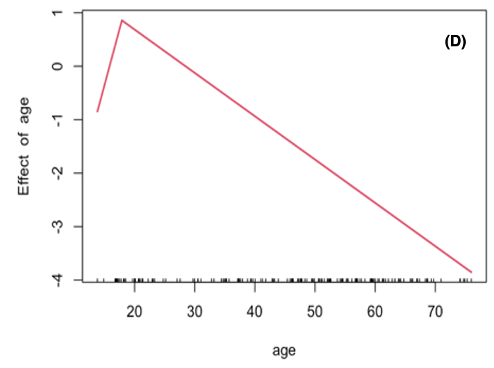


*(A) False-positive MRI for lateral meniscus tears. (B) False-negative MRI for lateral meniscus tears. (C) False-positive MRI for medial meniscus tears. (D) False-negative MRI for medial meniscus tears.*
